# 3D patterned stem cell differentiation using thermo-responsive methylcellulose hydrogel molds

**DOI:** 10.1038/srep29408

**Published:** 2016-07-06

**Authors:** Wonjae Lee, Jon Park

**Affiliations:** 1 Department of Neurosurgery, Stanford University, 300 Pasteur Drive, Stanford, CA 94305, USA; 2 Neurosciences Institute, Stanford University, 318 Campus Drive, Stanford, CA 94305, USA

## Abstract

Tissue-specific patterned stem cell differentiation serves as the basis for the development, remodeling, and regeneration of the multicellular structure of the native tissues. We herein proposed a cytocompatible 3D casting process to recapitulate this patterned stem cell differentiation for reconstructing multicellular tissues *in vitro*. We first reconstituted the 2D culture conditions for stem cell fate control within 3D hydrogel by incorporating the sets of the diffusible signal molecules delivered through drug-releasing microparticles. Then, utilizing thermo-responsivity of methylcellulose (MC), we developed a cytocompatible casting process to mold these hydrogels into specific 3D configurations, generating the targeted spatial gradients of diffusible signal molecules. The liquid phase of the MC solution was viscous enough to adopt the shapes of 3D impression patterns, while the gelated MC served as a reliable mold for patterning the hydrogel prepolymers. When these patterned hydrogels were integrated together, the stem cells in each hydrogel distinctly differentiated toward individually defined fates, resulting in the formation of the multicellular tissue structure bearing the very structural integrity and characteristics as seen in vascularized bones and osteochondral tissues.

The multilineage differentiation of stem cells is elaborately coordinated by multiple guidance cues and this tissue-specific patterned differentiation is the basis for the development, remodeling, and regeneration of the structure and functionality of multicellular tissues. There has been significant progress in stimulating *ex vivo* as well as *in vivo* expansion and differentiation of stem cells into functional progeny that could potentially regenerate the injured tissues^1^. Despite the wide range of the identified differentiation guidance cues, including the matrix elasticity^2^, the cell shape^3^, the cellular interaction^4^, nano-scale features^5^, and the mechanical stress^6^, the current endeavors toward stem cell fate control are mostly based on the identification of diffusible signal molecules and their receptor-mediated downstream signaling pathways. The majority of these investigations are performed using 2D cultures where the stem cell behaviors are monitored in response to the signal molecules diffusing freely in cell culture medium. Although this simple 2D experimental setup has facilitated elucidation of a broad range of signal molecules and their underlying mechanisms, there also arises a consequential need to develop a reliable platform to translate these fundamental findings into the 3D microenvironments and structures of the native tissues.

Hydrogels have been considered suitable as a scaffold material to provide structural and functional support equal to the native 3D tissues they replace due to their structural similarity to the extracellular components in the body^7^. Substantial interest has emerged in designing hydrogel networks that incorporate bioactive moieties, such as oligopeptides, small bioactive molecules, and functional groups, so that they can induce specific cell behaviors and stem cell differentiation into a diverse range of specialized cell types^8^. However, intact incorporation of these bioactive moieties into 3D hydrogel networks is technically challenging, not only because most of the biomolecules easily lose their therapeutic potency depending on ambient temperature and pH level or in the presence of any chemicals with proteolytic potential, but also because these chemical reactions oftentimes adversely affect the live cells present in the hydrogels. The incorporation process thus should be performed within a very narrow window of chemical reaction conditions to avoid the denaturalization of these moieties^9^ and the interference with the natural biochemical activities of the cells^10^. Another requirement hard to be met is that the concentration of each signal molecule should be maintained within therapeutic levels over prolonged time period until the stem cell differentiation reaches the terminal maturation stage. When the concentrations of the signal molecules decrease below the therapeutic level, it leads a population of stem cells to regress to a more undifferentiated state or to differentiate along alternative pathways^11^. These technical difficulties have forced many previous approaches toward 3D hydrogel scaffold design to be largely confined to incorporating only certain types of signal molecules^8^, although most of the unveiled pathways of lineage-specific differentiation in 2D cultures are regulated by complex interplays of multiple types of diffusible signal molecules.

Another important challenge in designing hydrogel scaffolds for stem cell-based tissue engineering is how to spatially control the differentiation signals in a way to recapitulate the polarized microenvironments of the native tissues and to induce the patterned stem cell differentiation^12^. This is because many stem cell behaviors in developmental, remodeling, and regenerative phases are induced by their responses to the specific spatial distribution of the signal molecules^12^. Therefore, enabling the patterned differentiation is vital to develop a native-like multicellular tissue structure which is in turn critical for its functionality. There has been growing interest in using photoconjugation or photocleavage method to generate polarized 3D environments^10^,^13^. These methods initiate photoreactions at specific regions of 3D hydrogels through temporospatial regulation of UV or visible light. Although they are notable technical advances, these methods are not optimal for guiding stem cell differentiation into a variety of specified lineages. In this work, we propose a cytocompatible design platform in which the patterned stem cell differentiation can be induced through regulation of the spatial distribution of diffusible signal molecules, leading to the formation of 3D multicellular tissue structures.

## Results

### Reconstitution of 2D culture conditions into 3D hydrogel

In this part of the study, we aimed to design a versatile 3D hydrogel platform in which the human mesenchymal stem cell (hMSC) fate control mechanisms identified in 2D cultures, especially those through diffusible signal molecules, could be directly translated and applied. We first examined how long the diffusible signal molecules should be present for the hMSC to reach the terminal maturation and thus yield maximum differentiation outcome in 3D hydrogel matrices. The interactions between multiple types of diffusible signal molecules are known not only to initiate the desired differentiation of the stem cells but also to direct them toward terminal maturation^11^. Once the terminal maturation occurs, the signal molecules would not significantly influence the differentiation outcome, and thus no longer be needed. We chose to investigate the osteo- and chondro-genesis since their differentiation outcomes in 3D hydrogel matrices could be evaluated simply by measuring the amounts of the synthesized calcium and glycosaminoglycan (GAG), respectively.

**Figure 1:**
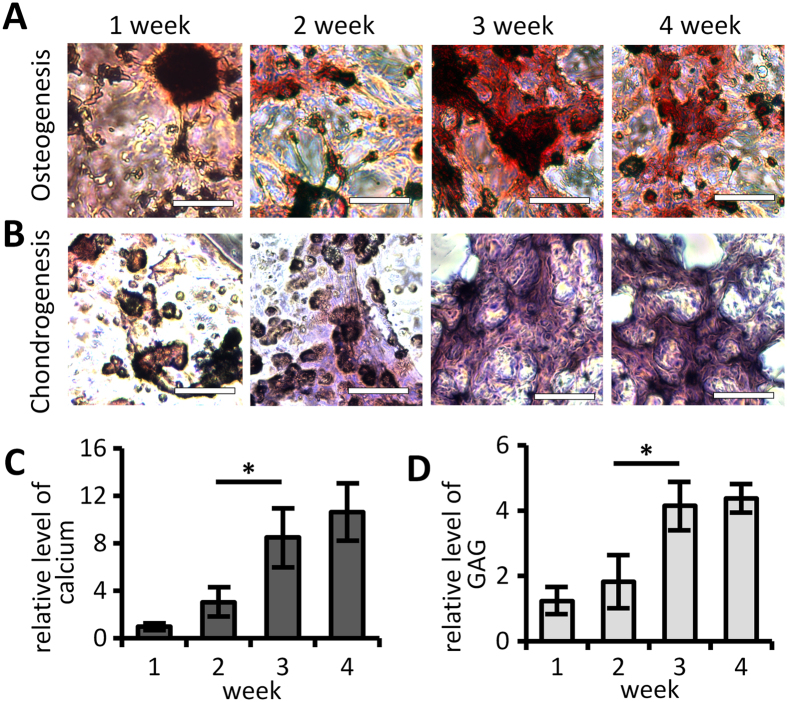
Incubation period for maximizing differential outcomes in hMSC-containing hydrogel. (**A**) The histological images of the osteogenic samples with Alizarin Red staining. The calcium deposition was stained as red. Scale bar: 150 μm. (**B**) The collagen in the chondrogenic samples was stained as pink by H&E staining. Scale bar: 150 μm. The differentiation outcomes, the calcium deposition for osteogenesis (**C**) and the GAG for chondrogenesis (**D**), were biochemically quantified. And their relative levels were calculated by taking the ratio between the differentiation outcome in each sample at specific time point and the outcome with 1-week incubation. The statistical significance is denoted as ‘*’(n = 3).

We incorporated hMSC in 3D hydrogel matrices and incubated them with the sets of the diffusible signal molecules identified in 2D cultures (Fig. 1). Over the course of four weeks, we removed the signal molecules from the hydrogel matrix sample groups at the designated time points and kept all sample groups in basal medium until the end of the four-week period at which point the outcomes of their differentiation were analyzed. For osteogenic differentiation, we observed that the amount of the synthesized calcium was significantly less when the signal molecules were removed after only one week or two weeks of incubation compared to longer incubation period (Fig. 1A,C). However, from the three-week-point on, there was no significant further increase in the amount of the synthesized calcium, implying that the osteogenic differentiation from hMSC reached its maximum around the third week of the co-incubation with the signal molecules. The similar pattern was shown in the samples for chondrogenesis (Fig. 1B,D). When the signal molecules were removed before three weeks, the synthesized amounts of glycosaminoglycan (GAG) were significantly less, but from the three-week-point on, there was no significant increase in the final outcome of the chondrogenic differentiation. These data match well with the data from the original 2D protocols where the maturation of hMSC differentiation also takes about three weeks in each study^14^,^15^. These results led us to reasonably assume that there was no significant difference in the required incubation period for the maturation of hMSC differentiation between the 2D and the 3D conditions. Thus we targeted to maintain the concentrations of the diffusible signal molecules in our main experiments up to three weeks.

**Figure 2:**
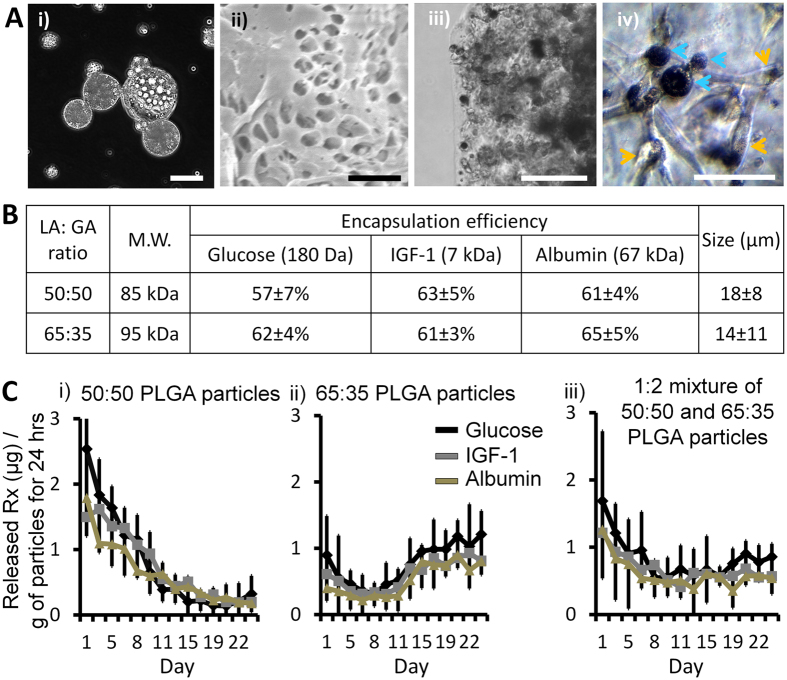
Sustained release of diffusible signal molecules through microparticles. (**A**) (i) The darkfield image of the drug-releasing microparticles (scale bar: 10 μm). (ii) The S.E.M. image of the microparticle cross-section (scale bar: 2 μm). (iii) The brightfield image of a hydrogel matrix containing the microparticles (scale bar: 400 μm). (iv) The phase contrast image of the drug-releasing particles (blue arrows) and hMSC (orange arrows) in a hydrogel matrix (scale bar: 50 μm). (**B**) Characterization of the microparticles encapsulating the blends of glucose, IGF-1, and albumin. ‘LA: GA ratio’ denotes the composition ratio of lactic acid (LA) units and glycolic acid (GA) units. ‘M.W.’ denotes the molecular weight of each PLGA. Drug encapsulation efficiency is the percentage of the actual amounts of each molecule encapsulated in the microparticles over their theoretical amounts added during the particle preparation. ‘Size’ represents the average diameter of each type of microparticles. (**C**) The releasing profiles of the three biomolecules from the microparticles. The 1:2 mixtures of 50:50 and 65:35 microparticles (iii) showed relatively continuous releasing rates compared to the individual microparticle types (i and ii) and was used to maintain the concentrations of the released signal molecules in the hydrogel matrices (n = 3).

As the medium for the sustained delivery of the signal molecules, we chose poly(lactic-co-glycolic acid) (PLGA) due to its biodegradability and biocompatibility^16^. Because many of the diffusible signal molecules are peptides or proteins, we encapsulated them into PLGA microparticles using the water-in-oil-in-water (w/o/w) method, known to be suitable for carrying easily-denaturing peptide or protein-based drugs without compensating their therapeutic efficacy^17^. Figure 2A shows the images of the PLGA microparticles (Fig. 2A(i) and (ii)) and the particles incorporated in a 3D hydrogel matrix together with hMSC (Fig. 2A(iii) and (iv)). Since the drug-releasing kinetics depend on the molecular weight of the encapsulated molecules, as a proof of concept, we encapsulated a blend of three biomolecules with low, medium, and high molecular weight respectively: glucose (180 Da), insulin-like growth factor I (IGF-1, 7 kDa), and albumin (67 kDa) (Fig. 2B). These molecules roughly covered the whole range of the molecular weights of the signal molecules in various 2D hMSC differentiation protocols (Table S1 in S.I.).

To obtain the sustained delivery over the targeted time period, we utilized the controlled drug-release technique^16^ where the releasing rate was controlled by modulating the mixture composition of the different types of the drug-releasing PLGA microparticles. We prepared different microparticle types by modulating the PLGA composition, from 50:50 (composed of 50:50 molar ratio of glycolide units and lactide units) to 65:35, to generate the composite drug-releasing profile with sustained release characteristic. The drug-releasing rate from 50:50 PLGA microparticles showed the early initial-burst followed by the gradual decrease (Fig. 2C(i)), while 65:35 PLGA microparticles had the gradually increasing release profiles (Fig. 2C(ii)). In order to grant a sustained release up to three weeks, we mixed the 50:50 and 65:35 PLGA microparticles in the ratio of 1 to 2 and obtained a relatively continuous release profile up to three weeks (Fig. 2C(iii)). The range of the therapeutic concentration levels of many biomolecules were quite broad, spanning a couple of orders of magnitudes between individual reports^18^,^19^, and so it could be reasonably assumed that the slight deviations observed in the releasing rates of the different signal molecules would not significantly impinge on the corresponding differentiation outcome.

### Inducing multiple types of hMSC differentiation within 3D hydrogel matrices

In order to examine the versatility of our approach, we chose protocols for multiple types of 2D hMSC differentiation such as adipogenesis^20^, and endothelial differentiation^21^ in addition to the osteogenesis^14^ and chondrogenesis^15^ protocols (Table S1 in S.I.). The reason that we preferred serum-free protocols was that they allowed us to control the concentration of the identified signal molecules in the microparticles so that the released amount could reach the therapeutic concentration level. We also expected that this approach would more reliably induce the spontaneous differentiation of stem cells than the serum-dependent protocols for many degenerative tissue lesions when implanted in the body. We encapsulated the blends of the identified diffusible signal molecules for each differentiation type into microparticles at five hundred times over the reported concentrations and incorporated them at 5% w/v within the hMSC-containing hydrogel matrices. For the case of endothelial differentiation, due to the scarcity of serum-free protocols in literature, we followed the serum-dependent protocol proposed by J. Oswald *et al*.^21^ and established the condition by adding the serum to the basal culture media. The cell-binding domains were incorporated in the hydrogel networks for all differentiation types, except for chondrogenesis.

**Figure 3:**
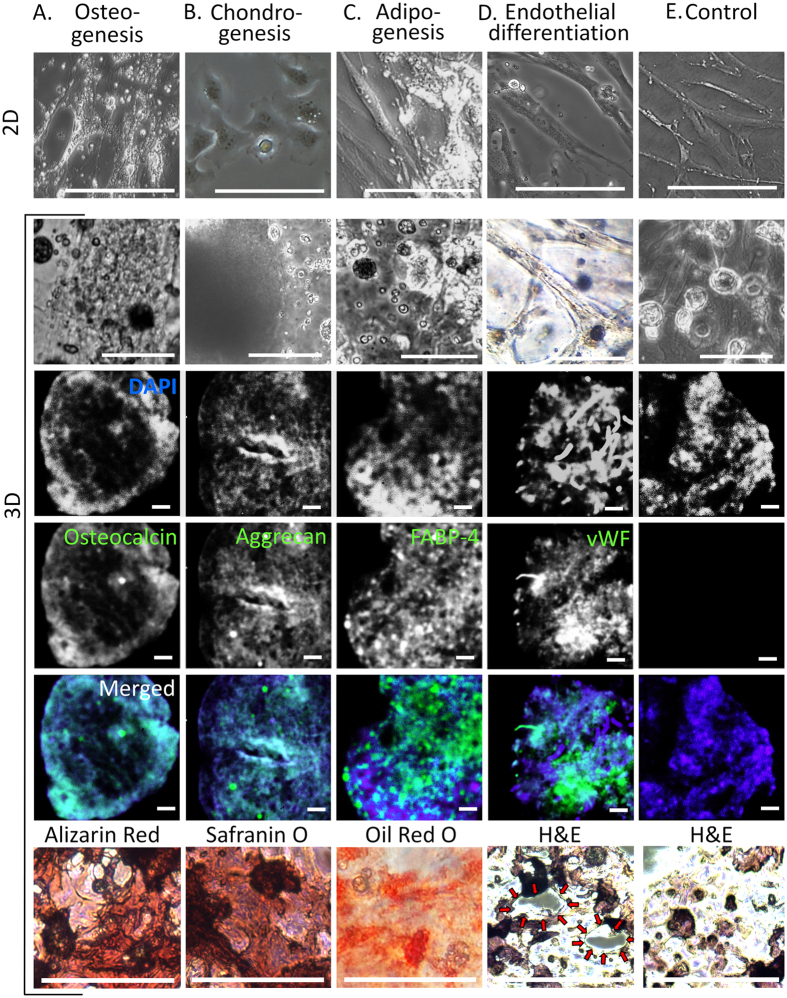
Multilineage differentiation of hMSC in 3D hydrogels. The phase contrast images in the first row show the morphological changes of hMSC cultured in 2D plates with the blend of the diffusible signal molecules with specific differentiation effects. (For the 2D image of chondrogenesis, due to the difficulty to induce chondrogenesis in traditional 2D culture plates, human primary chondrocytes were used.) Similar morphological changes were observed for the hMSC in 3D hydrogels in the presence of the microparticles encapsulating the same blends of the diffusible signal molecules (the phase contrast images in the second row). In the third to fifth rows, the targeted hMSC differentiation types in our 3D matrices were confirmed by the immunofluorescence of each differentiation marker. The third and fourth row show the fluorescent labeling of cell nuclei by DAPI and each differentiation marker, respectively. The fifth row is the merged images of the third and fourth rows. In the last row, histological images for each differentiation are presented. The red arrows in the image for the endothelial differentiation indicate the openings of the capillary-like structures. A set of the representative images from control experiments (without drug-releasing particles) is presented in the last column.

Under the influence of the released diffusible signal molecules for osteogenesis, the hMSC in 3D hydrogel formed aggregates and abundant opaque secretions, the morphology reported as mineralization in 3D hydrogel^22^ (Fig. 3A, the second row). We confirmed the osteogenesis by detecting one of the osteoblast maturation markers, osteocalcin (Fig. 3A, from the third to fifth rows). The synthesized calcium was stained as red with Alizarin Red (Fig. 3A, the last row). In the 3D hydrogel matrices aimed for chondrogenesis, we utilized the hydrogels without cell-binding domains. In the hydrogels destined for chondrogenesis, we observed the dense dark nodules (Fig. 3B, the second row) that had the similar morphology as in a previously reported chondrogenesis study^23^. These samples showed a positive stain for cartilage markers, aggrecan (Fig. 3B, from the third to fifth rows) and GAG (Fig. 3B, the last row), confirming successful chondrogenesis. The effect of the presence of the cell-binding domains on the chondrogenic and osteogenic outcomes is described in Fig. S1 in S.I. In both 2D and 3D hydrogel matrices aimed for adipogenesis, the shiny distinct areas previously reported as lipid accumulation during adipogenesis^20^ appeared (Fig. 3C, the first two rows). The subsequent analyses of these areas with the immunochemical staining for FABP-4 (Fig. 3C, from the third to fifth rows) and histological staining for lipids, the markers expressed in mature adipocyte cells, came out positive (Fig. 3C, the last row), confirming successful adipogenesis. In the 3D hydrogel matrices aimed for endothelial differentiation, the capillary-like tubular structure formation was observed (Fig. 3D, the second row), similar to the report of *in vitro* angiogenesis in hydrogel matrices^24^. We confirmed the endothelial differentiation by detecting a marker for mature endothelial cells, von Willebrand Factor (vWF) (Fig. 3D, the last three rows). In the cross-sectional image (Fig. 3D, the last row), the openings of capillary-like structures (red arrows) were observed. The control experiments for all types of differentiations were done with hMSC-containing hydrogel matrices cultured in the same conditions as the main experiments but without the microparticles and the signal molecules. A representative set of results from the control conditions is shown in Fig. 3E. We observed no significant difference in the differentiation outcomes across the control conditions. Additional images of the morphological changes of hMSC in the experimental conditions are shown in Fig. S2 in S.I.

### Cytocompatible casting process using thermo-responsive methylcellulose (MC) mold

**Figure 4:**
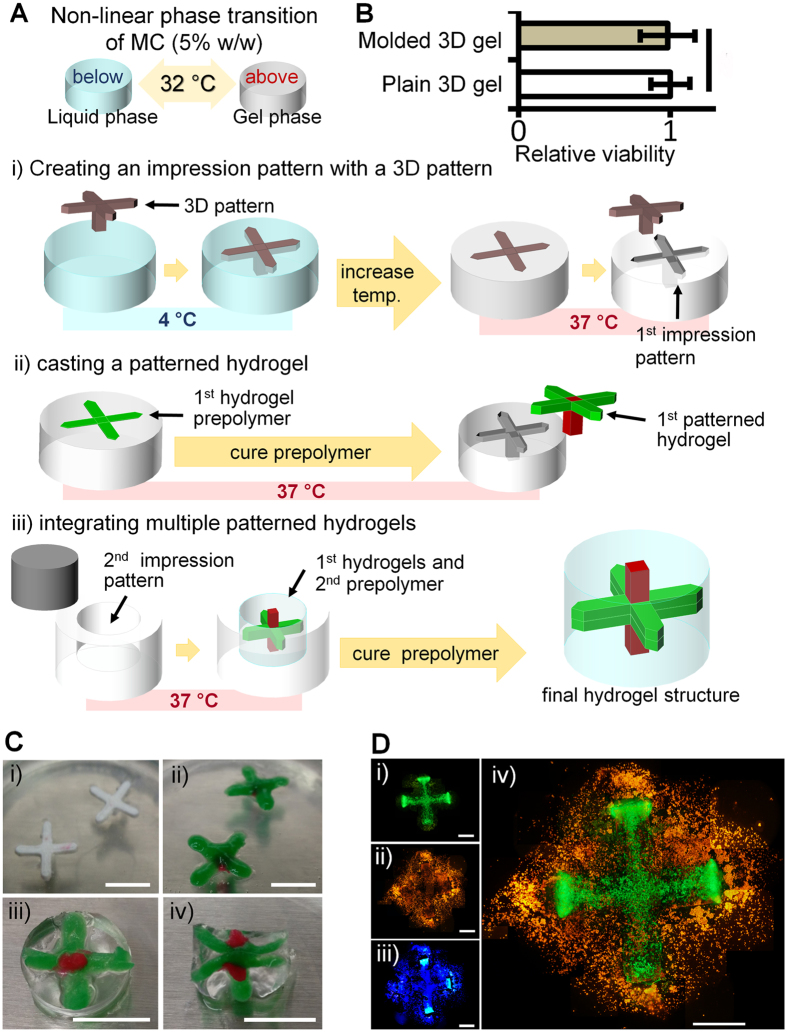
Thermo-responsive MC-based casting process. (**A**) Schematic illustration of MC-based casting process. 3D impression patterns were placed in the viscous MC solution at 4 °C and removed when the MC gelated at 37 °C. The hollow structure in the gelated MC was then filled with the hydrogel prepolymer which was subsequently cured into the corresponding 3D hydrogel structure. Finally, the hydrogel structures were integrated together in a bigger hydrogel matrix. (**B**) The cell viability was not compromised during the overall casting process (statically insignificant, n = 3). The relative viability was calculated by taking the ratio of the cell viability in the molded 3D hydrogels to the viability in the plain 3D hydrogels. (**C**) (i) An image of a representative 3D pattern on a gelated MC. (ii) An image of the hydrogel cured according to the 3D pattern shown in (i). (iii) and (iv) show the top and cross-sectional views of the final hydrogel structure. Green and red dyes were added into the hydrogels for presentation purposes. (**D**) The fluorescence images of a molded hydrogel. The hMSC were prestained as green (DiO) (i) and orange (DiI) (ii) and counter-stained with hoechst (iii). (iv) The merged image of (i and ii) (scale bar: 2 mm).

Due to its biocompatibility and sol-gel phase transition around body temperature, MC is now being widely used for a variety of biomedical applications such as drug delivery, wound healing, and tissue engineering^25^. The reversible gelation of MC arises from the enhanced inter-molecule interaction as the MC solubility in water decreases in higher temperature^26^. We utilized this thermo-responsivity as well as other well-documented physiochemical properties of MC^27^ to develop a MC-based mold for patterning hydrogels. The incident gelation, the emergence of the clusters of gel within the solution, occurs around 32 °C for 5% (w/w) MC solution^26^. The MC solution at room temperature and below has the viscosity of about 200 cP (mPa·s) and the gel has the dynamic viscosity of about 5,000 cP at 37 °C 26. In our experiments, the MC solution was viscous enough to hold 3D millimeter-scaled impression patterns and the subsequent gelation generated a reliable gel mold shaped as the impression patterns. We filled hMSC-containing hydrogel prepolymer into the MC mold of targeted shape and cured it at 37 °C in CO_2_ incubator. The overall casting process is described in Fig. 4A,C. The overall casting process was performed around body temperature within a short time frame (about 1 hour) leaving no adverse effect on the cell viability (Fig. 4B). Figure 4D shows the molded hydrogels through this casting process with the encapsulated hMSC.

### Reconstruction of multicellular 3D tissues

**Figure 5:**
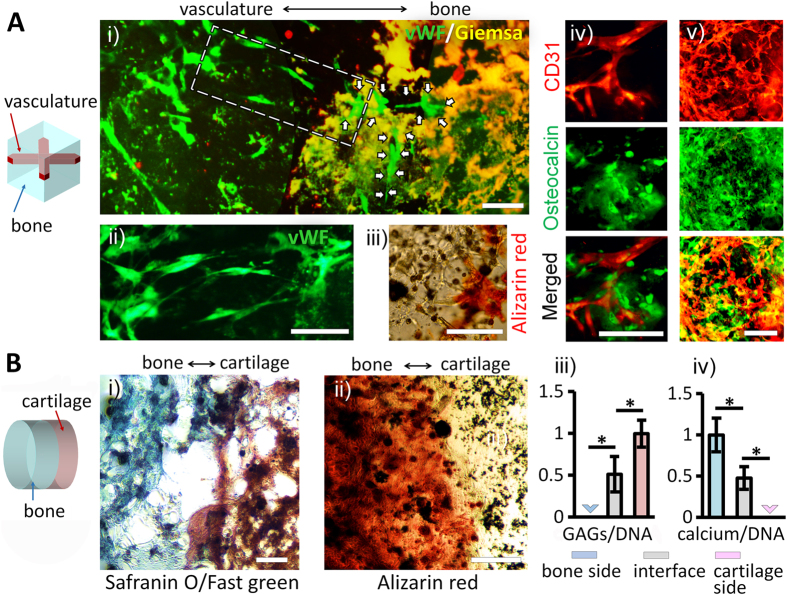
Reconstruction of multicellular 3D tissues. (**A**) Engineered vascularized bone. The hydrogel designated for endothelial differentiation was shaped into a cross and then integrated within the hydrogel matrix designated for osteogenesis. (i) The fluorescence image of the interface between the hydrogel regions targeted toward endothelial differentiation and toward osteogenesis. The endothelial cells and the calcified region were identified by staining with anti-vWF (green) and with Giemsa (yellow), respectively. White arrows indicate the endothelial cells penetrated into the bone structure. (ii) A magnified image around the dashed area in (i). (iii) The brightfield image of the interface. The calcified bone region was stained as red with Alizarin red. (iv) and (v) show the fluorescence images of the endothelial cells (red) and osteoblasts (green) immunohistochemically stained for CD31 and osteocalcin, respectively (scale bar: 200 μm). (**B**) Engineered osteochondral tissue. The two hydrogel layers, each designated for osteogenesis and chondrogenesis respectively, were stacked together. Histological images of the engineered osteochondral interface were obtained with Safranin O/Fast green stain (proteoglycan: red, background proteins: green) in (i) and Alizarin red (calcium: red) in (ii) (Scale bar: 250 μm). The quantitative analyses of the bone-side, the interface, and the cartilage-side segments were performed by measuring the amount of GAG (iii) and calcium (iv) deposition normalized by the total DNA amounts (n = 3). The statistical significance and the data below detectable levels are denoted as ‘*’ and ‘v’, respectively.

By integrating distinctly patterned hydrogels, each with hMSC differentiating toward individually targeted fate, it was possible to set up the polarized microenvironments optimal for generation of the multicellular tissue structure. We chose the vascularized bone and the osteochondral tissue as our target tissue structures to build with this method. For the vascularized bone, the hydrogel with hMSC differentiating toward endothelial cells was molded into a cross shape and then integrated within the surrounding hydrogel matrix designated for osteogenesis (Fig. 5A). The osteochondral tissue was reconstructed by stacking two hydrogel layers, each designated for osteogenesis and chondrogenesis respectively (Fig. 5B). We incorporated the same sets of the diffusible signal molecules for triggering each differentiation type as previously used (Table S1 in S.I.). After four weeks of incubation, we carried out analyses on these multicellular tissue structures and observed the expected characteristics of the targeted native tissues in our reconstructed tissues.

In the engineered vascularized bones, the endothelial differentiation and osteogenesis were induced, each in the distinct, targeted region as guided by the hydrogel structure (Fig. 5A). We confirmed the targeted differentiation outcome in each region and analyzed the interface between the two regions using immunochemistry. We first identified the mineralization by Giemsa stain (Fig. 5A(i)) that emitted both green and red fluorescence^28^ and visualized the calcified area as yellow. Interestingly, the differentiated endothelial cells sprouted toward and penetrated into the mineralized, bone region (Fig. 5A(i) and (ii)). Figure 5A (iii) also shows the formed capillary-like tubes in vascular region penetrating into the calcified region. The migration of the endothelial cells into the osteogenic area was confirmed by specific markers for each type of the cells, CD31 for endothelial cells and osteocalcin for osteoblast respectively (Fig. 5A(iv) and (v)). It was likely that the osteogenic signals released in the bone region of the hydrogel structure, especially the bone morphogenic protein-2^29^, attracted the migration of the endothelial cells into the osteogenic area, although more extensive investigation is required to confirm. Because the vascularization plays a pivotal role in bone development and fracture repair^30^, these results bear especially significant implications for bone regenerative medicine.

For the case of the engineered osteochondral tissue, in contrast to the extensive interfacial interaction between vasculature and bone regions shown in the vascularized bone, we observed a well-defined boundary between the bone and cartilage regions, the characteristic structure of the native osteochondral interface (Fig. 5B). And at the interface of our engineered osteochondral tissue, two distinct crossing gradients of GAG (stained with Safranin O) and calcium (stained with Alizarin red) were observed (Fig. 5B(i) and (ii)), similar to the morphology found in the osteochondral interface *in vivo*^31^. These histological observations were in accord with the quantitative analyses of the amounts of GAG and calcium found in the bone-side, interface, and cartilage-side segments; the amount of GAG in each segment was inversely proportional to the amount of calcium (Fig. 5B(iii) and (iv)). In contrast, the molded samples without drug-releasing microparticles did not show these differential behaviors at the interfaces (Fig. S3 in S.I.). These results demonstrate that our approach was successful in reconstructing hetero-cellular tissue structures in 3D hydrogel matrix.

## Discussion

In this study, we proposed a versatile and accessible design platform that could directly translate the 2D culture conditions for hMSC differentiation into 3D environment for the reconstruction of 3D multicellular tissues. We first demonstrated that the 2D differentiation conditions could be reconstituted within 3D hydrogels simply by incorporating the corresponding sets of the diffusible signals identified in 2D conditions through drug-releasing microparticles. Then we proposed a novel cytocompatible casting process in which these hydrogels could be molded into specific 3D configurations. The overall casting process involved minimal cellular stress since the mild temperature changes for MC phase transition (between the body and room temperatures) was the only adverse factor for the environment of the incorporated cells. This approach was well suited for inducing polarized 3D microenvironments required for the development of multicellular tissue structures, because it could localize the diffusible signals specifying the differentiation fate into biodegradable microparticles and deliver them to the very sites of the stem cells. From these polarized microenvironments established through the spatial regulation of the released signals, the targeted stem cell differentiation with the specified 3D patterns successfully emerged. The patterned stem cell differentiation achieved this way formed the 3D multicellular structure of the targeted tissues, the vascularized bone and the osteochondral tissue, bearing practical implications for clinical application. The migration of endothelial cells into the neighboring bone segment shown in our engineered tissue is especially promising, because any engineered tissue would require functional vascular system to immediately support metabolic activities of the incorporated cells after being implanted, which has been considered as one of the major challenges in 3D tissue engineering^32^. The successful reconstruction of the osteochondral interface also has important clinical implications for regenerating cartilage, since its degeneration process is often coupled with the dynamics in the adjacent subchondral bone^31^.

The approach proposed here can contribute to close the “bench-to-bedside” gap, enabling the translation of the many discoveries on stem cell fate decision in 2D experimental conditions into the regeneration of 3D multicellular tissues. Thus far, the engineering approaches to reconstruct stem cell niches in 3D scaffolds have largely focused on identifying the microenvironment and cues for stem cell fate decision that are only revealed in 3D conditions, distinct from those already found in 2D conditions. These efforts revealed that the topology and mechanical properties of the 3D scaffolds, as well as the spatio-temporal profiles of delivering the differentiation cues into the scaffolds all played an important part in specifying stem cell fates^12^. In traditional 2D cultures, however, many types of differentiation are reliably triggered by the diffusible signals alone, without the factors identified in 3D conditions. 2D cultures are currently the most widely used experimental setup in biomedical research, leading to the identification of a broad range of signal molecules for stem cell differentiation and their receptor-mediated downstream signaling pathways in 2D conditions. These extensive findings based on 2D cultures, however, have not been effectively utilized for clinical applications, especially for stem-cell based tissue regeneration, because of the lack of proper experimental platform to translate the 2D conditions into more native-like 3D environment.

Our approach addresses and satisfies this critical need for a 3D tissue design platform compatible with 2D experimental findings. In our 3D hydrogel matrices, the multilineage stem cell differentiation was successfully controlled exactly the same way as in 2D culture conditions with the same diffusible signal molecules. With the feature of spatio-temporally controlled delivery of signal molecules, our approach is versatile, immediately applicable to inducing the patterned stem cell differentiation targeting other tissue structures or to establishing microenvironments for many other biological processes governed by diffusible signal molecules. Our approach can also serve as a useful platform for further investigation on the interplay between diffusible signal molecules and other important microenvironmental cues for stem cell differentiation.

Our approach is also highly accessible, with its major components being the methodologies already utilized in many traditional biomedical laboratories. For example, the localization of diffusible signal molecules in hydrogel matrices was achieved through one of the most well-established drug-delivery techniques, the double emulsion process, using a standard homogenizer. Also, the cytocompatible 3D casting process was developed based on the thermo-responsivity of MC gel, commonly available in biomedical laboratories. With its versatility and accessibility, our translational platform proposed here will contribute to expedite the applications of the key findings in 2D stem cell research to regeneration of 3D multicellular tissues.

## Materials and Methods

### Preparation of drug-releasing microparticles

The drug-releasing PLGA microspheres were prepared through the double emulsion process (water-in-oil-in-water (w/o/w)) as described previously^16^. Briefly, 50:50 or 65:35 PLGA (LACTEL) were dissolved in the non-polar organic solvent, dichloromethane (DCM), at 20% (w/v). For the preliminary investigation of the multi-molecule release patterns, D-glucose (Sigma), IGF-1 (R&D Systems), and albumin from bovine serum (BSA, Sigma-Aldrich) were dissolved in PBS at 100 μg/ml. For the main experiments, blends of the identified diffusible signal molecules (Table S1 in S.I.) were dissolved in PBS at five hundred times concentration than the reported concentrations. Each type of the prepared drug solutions was added to the PLGA solution at the one to nine ratio and then emulsified for 1 min at approximately 30,000 rpm using a homogenizer (Pro Scientific). The emulsion was poured into 1000 times its volume of ice-cold PBS. The solution was stirred at 10,000 rpm for 10 min using a homogenizer and then moved to a magnetic stirrer for continuous stirring to allow DCM to evaporate overnight. The resulting solid microspheres were collected by centrifugation. The diameters of the microparticles were obtained through the analysis of the brightfield microscopic images using AxioVision image software (Carl Zeiss).

Drug encapsulation efficiency was calculated by comparing the actual amounts of D-glucose (Sigma-Aldrich), IGF-1 (R&D System), and bovine serum albumin (BSA, Sigma-Aldrich) encapsulated in the microparticles over the theoretical amount of each molecule during the particle preparation, as described previously^16^. PLGA microparticles were dissolved in acetone and the precipitate was collected by centrifugation. The collected precipitate was dissolved in 1 ml of 1 M sodium hydroxide (NaOH) and the solution was neutralized with hydrochloric acid and NaOH by use of a pH meter (Corning). After diluting the solution to the measurable range of each measurement tool, the total amount of each molecule type was determined by a glucose meter (Germaine Laboratories), an IGF-1 ELISA kit (Enzo Life Sciences), and Albumin Blue Fluorescent Assay Kit (Active Motif) (n = 3) respectively. For the drug-releasing profiles, the drug-releasing microparticles were incubated in PBS and the supernatants were collected at designated time points and stored at −20 °C until analysis.

### Construction of 3D hydrogel matrices

We prepared the hydrogels based on matrix metalloproteinases (MMPs)-sensitive polyethylene glycol (PEG)^33^. 4-arm-PEG-Vinylsulfone (10 or 20 kDa, JenKem Technology USA Inc.) was dissolved in triethanolamine buffer (0.3 M, pH 8.0) at the final concentration of 15% (w/v). For the hydrogel networks with cell-binding domains, the peptides containing arginylglycylaspartic acid (RGD) sequence (Ac-GCGYGRGDSPG-NH2, American Peptide Company) were also added at 0.3 mM final concentration. After 10 min, the MMP substrates (Ac-GCRDGPQGIWGQDRCG-NH2, American Peptide Company) were added to the solution at the stoichiometric amounts (30 mM and 15 mM for 10kDa and 20kDa 4-arm-PEG-Vinylsulfone, respectively). And hMSC suspension was added into each hydrogel prepolymer at the final concentration of 20 × 10^6^ cells/ml with the drug-releasing microparticles at the final concentration of 5% (w/v). The final prepolymer solution was loaded between glass slides (VWR) separated by a Teflon spacer (~1 mm thickness) and incubated in a CO_2_ incubator at 37 °C with 5% CO_2_ for 45 min. The cured hydrogels were subsequently washed with PBS and then cultured in ultra-low attachment multiwell plates (Corning) with Mesenchymal Stem Cell Basal Medium (Thermo Scientific). The elastic shear modulus of the same hydrogel was reported as about 3.8 kPa^34^. For the endothelial differentiation, 2% (v/v) of fetal calf serum (Remel™, Thermo Scientific) was added to the basal medium. Only half of the medium was gently replaced every three days in order to minimize the loss of the released signal molecules diffusing out from the hydrogel matrices.

### MC-based casting process

Methylcellulose (Methocel^®^ A15 LV, Dow Chemical) solution (5% (w/w) in dH_2_O) was prepared by adding a designated amount of MC powder in distilled water and leaving it at 4 °C overnight. The 3D impression patterns (eMachineShop) built with a 3D printer were presoaked in mineral oil (Sigma) for a few hours so that the absorbed oil on the surface could help easy removal of the patterns from the MC mold. Then we placed the patterns in the clear and viscous MC solution and left them at a humidified CO_2_ incubator allowing for the MC solution to equilibrate to the gelation temperature, 37 °C, and form an opaque gel. When the patterns were removed from the MC gel, leaving the MC mold with the 3D configurations of the patterns, we filled the mold with the final hydrogel prepolymer designated for hMSC differentiation into specific lineage. After they were placed in a CO_2_ incubator at 37 °C to cure the prepolymer, the MC mold was left inside of a laminar flow hood at room temperature and the patterns were removed as the MC mold turned into liquid. For the construction of multicellular tissues, hydrogels with specified configurations were integrated together as described in Fig. 4A.

### Statistical analysis

Statistical analyses were performed by One-way ANOVA with Bonferroni-Holm post-hoc test. The results from these analyses are reported as the mean and the standard deviation of the mean. A confidence level of 95% was considered significant.

## Additional Information

**How to cite this article**: Lee, W. and Park, J. 3D patterned stem cell differentiation using thermo-responsive methylcellulose hydrogel molds. *Sci. Rep.* **6**, 29408; doi: 10.1038/srep29408 (2016).

## Supplementary Material

Supplementary Information
