# Supplementary material for: 3D patterned stem cell differentiation using thermo-responsive methylcellulose hydrogel molds
Source: Sci Rep. 2016 Jul 6;6:29408. doi: 10.1038/srep29408 (PMC4933913; doi:10.1038/srep29408)
Supplement: Supplementary Information [file srep29408-s1.pdf]

Supporting Information

3D patterned stem cell differentiation using thermo-responsive methylcellulose hydrogel molds

W. Lee [1,2,*], J. Park [1,*]
[1] Department of Neurosurgery, Stanford University, 300 Pasteur Drive, Stanford, CA 94305, USA
[2] Neurosciences Institute, Stanford University, 318 Campus Drive, Stanford, CA 94305, USA
* Corresponding authors, E-mail: wjh@stanford.edu (W. Lee), jonpark1@stanford.edu (J. Park)

CONTENTS

Materials and Methods
Table S1. The sets of the diffusible signal molecules used for specific hMSC differentiation.
Figure S1. The effect of the presence of the cell-binding domains on the chondrogenic outcomes in 3D hydrogel matrices.
Figure S2. Additional brightfield images of various types of hMSC differentiation in 3D hydrogel matrices.
Figure S3. The negative control conditions with molded hydrogel matrices but without drug-releasing microparticles.

MATERIALS and METHODS

Cell Culture

Human bone marrow mesenchymal stem cells (hMSC) were purchased from Invitrogen or Thermo Scientific and expanded in a growth medium (the mesenchymal stem cell basal medium (Thermo Scientific) with 10% (v/v) stem cell growth supplement (Thermo Scientific)) in a CO_2 incubator at 37 °C with 5% CO_2. The cells at passage four or five were used.

Immunofluorescence

The hydrogel matrices targeted for various types of stem cell differentiation were fixed at 4% paraformaldehyde solution for 15 min at room temperature and washed twice with ice-cold PBS. Then the samples were incubated for 10 min in PBS containing 0.25% Triton X-100 (Sigma-Aldrich) and washed with PBS three times for 5 min. After incubated with 1% BSA (Sigma-Aldrich) in PBST (0.1% Tween-20 (Sigma-Aldrich) in PBS) for 30 min, each sample was added to the corresponding primary antibodies in 1% BSA in PBST in a humidified cell incubator for 1 hr. The antibodies for human osteocalcin (R&D System), aggrecan (R&D System), FABP-4 (R&D System), and vWF (or CD31, Sigma-Aldrich) were used for the hydrogel matrices aimed for osteogenesis, chondrogenesis, adipogenesis, and endothelial differentiation respectively. After the solutions with the antibodies were removed, the samples were washed three times in PBS for 5 min each. The samples were then incubated in the fluorescin (FITC) or NL577-conjugated secondary antibody solutions in 1% BSA for 1 hr at room temperature in darkness. The solutions were removed and the samples were washed three times in PBS for 5 min each. And they were counter-stained with DAPI for 1 min and rinsed with PBS. After the samples were mounted between a glass slide and a coverslip with a drop of mounting medium, the fluorescence images were obtained with a confocal microscope or an inverted fluorescence microscope.

Histological Staining

All samples for histological staining were prepared with the cryostat sectioning (LEICA). For Alizarin Red Staining (LIFELINE), the samples were added to absolute ethanol for 30 min and then replaced to 2% Alizarin Red Stain Solution for 15 min at room temperature. They were rinsed with dH_2O and mounted between glass slides for imaging at a microscope. For Hematoxylin and Eosin (H&E) Staining (ScyTek Laboratories), the samples were applied to Hematoxylin solution for 5 min and rinsed twice with dH_2O. The samples were incubated for 15 seconds in Bluing Reagent and rinsed twice with dH_2O. After dipped for 15 seconds in absolute ethanol and rinsed twice with dH_2O, the samples were added to Eosin Solution for 3 min and rinsed with absolute ethanol three times before mounted for imaging. For Safranin O / Fast Green Staining (ScienCell), the samples were added to 0.1% Fast Green Solution for 10 min and rinsed with 1% Acetic Acid for 15 seconds. And they were incubated in 0.1% Safranin O staining solution for 30 min and rinsed with absolute ethanol three times before mounted for imaging. For Giemsa staining (Fluka), the samples prepared for the immunofluorescence of endothelial cells were immersed in Giemsa working

solution (1:20 diluted) for 45 min and rinsed with dH_2O before mounted for imaging. For Oil Red O staining (LifeLine), the samples were added to propylene glycol for 2 min, incubated in Oil Red O solution for 6 min, rinsed with propylene glycol (80% v/v in distilled dH_2O) for 1 min and with dH_2O twice, incubated in Hematoxylin for 2 min, and then rinsed with dH_2O twice before mounted for imaging.

Glycosaminoglycan (GAG) assay

Sulfated GAG content was measured using Blyscan sulfated GAG assay kit (Biocolor Ltd., Carrickfergus) according to the manufacturer's protocol. Briefly, each segment of the hydrogel matrices was homogenized (Pro Scientific Inc.), digested in 500 µl of 0.1 mg/ml pepsin in 0.1 M HCl overnight at room temperature, and neutralized with 1M NaOH. 100 µl of the digested solution was mixed with 250 µl Blyscan dye and shaken for 30 min. After centrifuging at 15,000 × g for 10 min, the precipitate was collected and added to the 600 µl dissociation reagent for 10 min to release the bound dye. The absorbance was then read at the wavelength of 656 nm and the actual amount of GAG was determined by the standard curve (the amount of GAG as a function of absorbance). The standard curve was generated with the standard samples provided in the kit.

Calcium quantitation assay

We analyzed the mineralization in the hydrogel matrices by measuring the amount of calcium with Osteogenesis Quantitation Kit (Millipore) according to the manufacturer's protocol. Briefly, each segment of the hydrogel matrices was added to 10% acetic acid in a microtube, homogenized (Pro Scientific Inc.), and shaken for 30 min. After heated at 85 ºC for 10 min, the microtube was transferred to iced water for 5 min and centrifuged at 20,000 × g for 15 min. With a pH meter (Corning), the pH of the supernatant was ensured to fall within the range of 4.1- 4.5. The absorbance was read at the wavelength of 405 nm and the actual amount of calcium was determined by the standard curve generated with the standard samples in the kit.

DNA Quantitation

The quantitation of DNA content was performed with a fluorescent dye, bisBenzimide. Each segment of the hydrogel matrices was homogenized (Pro Scientific Inc.) and added to 1 µg/ml bisBenzimide solution (Sigma-Aldrich) diluted by 10 × Fluorescent Assay buffer (Sigma-Aldrich) and water (Molecular Biology Grade, Fisher Scientific). Then the fluorescent intensity was read at the excitation wavelength of 360 nm and the emission wavelength of 460 nm. The actual DNA amount was determined by the standard curve prepared by DNA standard (1 mg/ml solution of calf thymus DNA, Sigma-Aldrich).

Viability test

Relative cell viabilities of samples were measured by the AlamarBlue® assay (Invitrogen). Briefly, 10 % (v/v) AlamarBlue reagent solution in the cell culture medium was added to each sample for 2 hours and 100 µl of each supernatant was collected into a 96 well plate. And the fluorescence of each sample was measured at the excitation wavelength at 545 nm and the emission wavelength at 590 nm. The fluorescence differences in the two wavelength were calculated. The relative viability was calculated by comparing the following value of each sample:

(fluorescence difference in the supernatant) – (fluorescence difference in a negative control, 10% AlamarBlue solution)

This method of measuring the cell viability in 3D hydrogel samples did not show statistical difference from the method of manual counting using Live/Dead staining (Invitrogen) [35].

Table S1.

	Soluble signal molecules			Reference
	name	amount	M.W.	
Osteogenesis	Dexamethasone<br>Ascorbic acid<br>β-glycerophosphate<br>Bone morphogenic protein-2	10 nM<br>300 μM<br>5 mM<br>100 nM	430 Da<br>180 Da<br>310 Da<br>26 kDa	Hildebrandt et al. [14]
Chondrogenesis	Dexamethasone<br>Ascorbic acid<br>Transforming growth factor-β<br>Insulin<br>Transferrin<br>selenious acid<br>Bone morphogenic protein-6	100 nM<br>200 μM<br>230 pM<br>1.7 μM<br>70 nM<br>54 nM<br>30 nM	430 Da<br>180 Da<br>44 kDa<br>5.8 kDa<br>80 kDa<br>130 Da<br>16 kDa	Fernandes et al. [15]
Adipogenesis	Insulin<br>3-isobutyl-1-methylxanthine<br>Transferrin<br>Triiodothyonine<br>Cortisol	66 nM<br>500 μM<br>130 nM<br>200 nM<br>30 μM	5.8 kDa<br>220 Da<br>80 kDa<br>660 Da<br>360 Da	van Harmelen et al. [20]
Endothelial differentiation	Fetal calf serum<br>Vascular endothelial growth factor	2% (v/v)<br>2.4 pM	N/A<br>21 kDa	Oswald et al. [21]

Table S1. The sets of the diffusible signal molecules used in our experiments for specific hMSC differentiation

Figure S1.

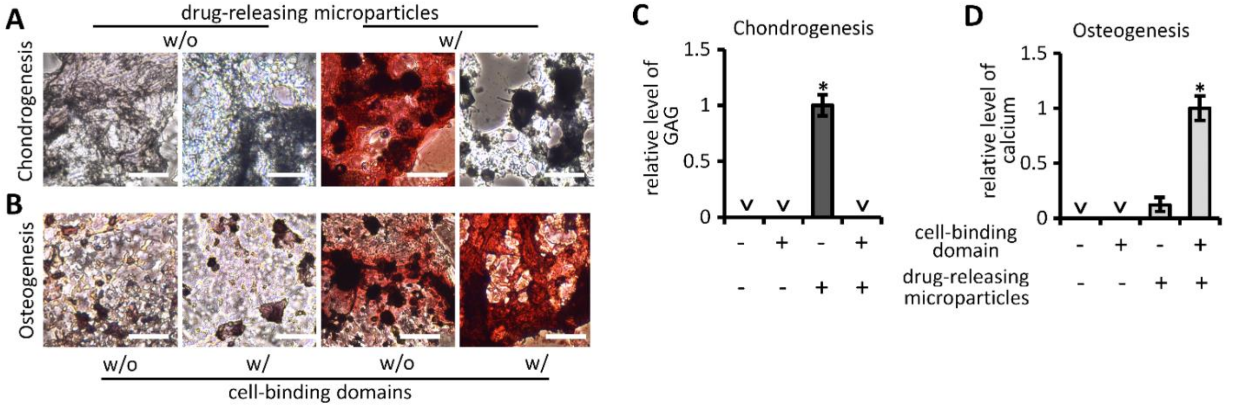

Fig. S1. The optimal condition for chondrogenesis and osteogenesis with respect to an immobilized cue (the cell-binding domains) was determined. Histological images of hMSC-containing hydrogel matrices are shown in **A** for chondrogenesis stained by Safranin O stain (GAG: red) and **B** for osteogenesis stained by Alizarin Red Stain (calcium: red). Scale bar: 150μm. Quantitative analyses on the differentiation outcomes confirm the results seen in the histological images that the incorporation of the cell-binding domains interferes the chondrogenesis (**C**) while the osteogenesis is critically dependent on the presence of the cell-binding domains (**D**). The statistical significance and the data below detectable levels are denoted as '*' as 'v', respectively (n=3).

Figure S2.

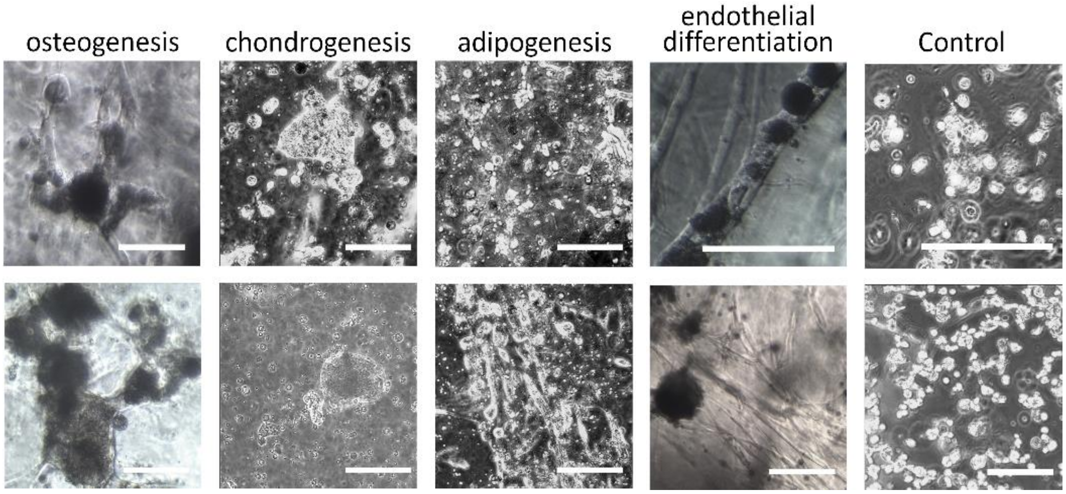

Fig. S2. Additional brightfield images of hMSC morphological changes during various types of differentiation process in 3D hydrogel matrices induced by the corresponding signal molecules released from microparticles. Scale bar: 250 μm.

Figure S3.

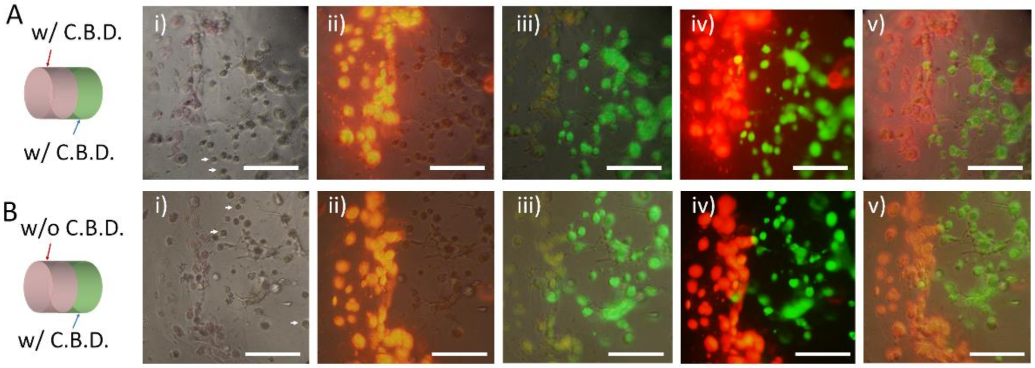

Fig. S3. The negative control conditions in which the hMSC cells were incubated in the molded hydrogel matrices prepared through MC casting process but without drug-releasing microparticles. The hMSC without the drug-releasing microparticles showed no differential behavior compared to the case with the drug-releasing microparticles (see **Fig. 3**, **Fig. 5**, and **Fig. S2**). Both hydrogel layers in **A** were prepared with the hydrogel networks with cell binding domains (C.B.D.). In **B**, one layer (with the prestained hMSC as green by DiO) contained C.B.D. in the hydrogel network, while the other layer (with the prestaiend hMSC as red by DiD) was without C.B.D. For both **A** and **B**, the images in the first column (**i**) show the brightfield images of hMSC at the interfaces of the hydrogel layers. To show these interfaces more clearly, columns (**ii**)-(**v**) present the merged images of the brightfield image in (**i**) and the fluorescence image of DiD and DiO. (**ii**) The brightfield image and the fluorescence image of DiD merged. (**iii**) The brightfield image and the fluorescence image of DiO merged. (**iv**) The fluorescence image of DiD and DiO merged. (**v**) The brightfield image and the fluorescence image of DiD and DiO altogether merged. Because we mostly utilized serum-free differentiation protocols, in these negative control samples without any signal molecules nor serum, some of the incorporated hMSC did not thrive as well as in the experimental conditions, showing typical morphological signs of cell death such as cell rounding, cell shrinkage and membrane blebbing (white arrows). Scale bar: 200 μm.
